# Fundraising and vote distribution: A non-equilibrium statistical approach

**DOI:** 10.1371/journal.pone.0223059

**Published:** 2019-10-30

**Authors:** Hygor P. M. Melo, Nuno A. M. Araújo, José S. Andrade

**Affiliations:** 1 Centro de Física Teórica e Computacional, Universidade de Lisboa, Lisboa, Portugal; 2 Instituto Federal de Educação, Ciência e Tecnologia do Ceará, Avenida Des. Armando de Sales Louzada, Acaraú, Ceará, Brazil; 3 Departamento de Física, Faculdade de Ciências, Universidade de Lisboa, Lisboa, Portugal; 4 Departamento de Física, Universidade Federal do Ceará, Fortaleza, Ceará, Brazil; Consejo Nacional de Investigaciones Cientificas y Tecnicas, ARGENTINA

## Abstract

The number of votes correlates strongly with the money spent in a campaign, but the relation between the two is not straightforward. Among other factors, the output of a ballot depends on the number of candidates, voters, and available resources. Here, we develop a conceptual framework based on Shannon entropy maximization and Superstatistics to establish a relation between the distributions of money spent by candidates and their votes. By establishing such a relation, we provide a tool to predict the outcome of a ballot and to alert for possible misconduct either in the report of fundraising and spending of campaigns or on vote counting. As an example, we consider real data from two proportional elections with more than 6000 candidates each, where a detailed data verification is virtually impossible, and show that the number of potential misconducting candidates to audit can be reduced to less than ten.

## Introduction

In an effort towards fair electoral processes, regulations and reforms are constantly on the agenda of many countries around the world [1]. To avoid that the decision-making process is dominated by wealth and influence, the most pertinent processes to legislate are arguably fundraising and spending [2]. Different countries have different rules, but in general, candidates and parties are the ones that report on the financial details of their own campaigns, what raises obvious doubts over the veracity of the reported data. As the number of collected votes correlates with the money spent in the campaign [3], establishing a quantitative relation between the distribution of votes and financial resources among the candidates is instrumental to raise flags about possible misconduct.

Within some regulated boundaries, several individuals or institutions can contribute financially to a campaign. The value of the contribution is very subjective, depending on their interests and on the economic and political conjecture [4–7]. Thus, predicting the distribution of funds raised and money spent in a campaign from “first principles” is likely a hopeless endeavor, challenging the verification of the reported data. In sharp contrast, the distribution of votes among candidates is well studied. It is known to differ for proportional and plural elections, and to depend on the country, number of candidates, and money spent in campaigns [8–13]. Different models were developed to explain this distribution [3, 14–19] as well as methodologies to identify vote-counting irregularities [20–25]. Here we propose an approach based on the Shannon entropy maximization and Superstatistics to derive a relation between the distribution of financial resources declared by candidates and the distribution of their votes in proportional elections.

## Results

**Fig 1:**
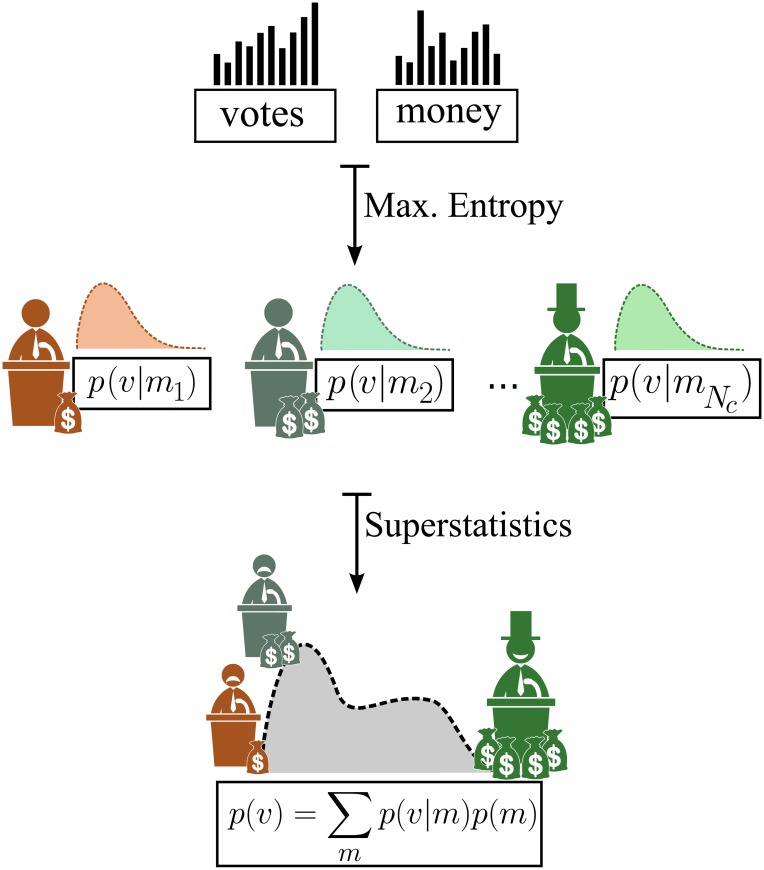
By employing the principle of maximum entropy under the constraints of a fixed number of voters and candidates, we derive the conditional probability *p*(*v*|*m*_*i*_), that a candidate *i* receives *v* votes, provided that *i* spends an amount of money *m*_*i*_ in the campaign. Since the amount of money spent usually differ from candidate to candidate, the final distribution of votes should depend on the distribution of money spent. A formalism based on Superstatistics [26] is then used to establish a relation between these two distributions.

Given a certain amount of money *m*_*i*_ spent by a candidate *i* in the campaign, the conditional probability for *i* to receive *v* votes is *p*(*v*|*m*_*i*_). Since the money spent is heterogeneously distributed among candidates, the probability *p*(*v*) that a candidate receives *v* votes is given by,

$$\begin{array}{c} p\left(v\right)=\sum_{m=0}^{m_{\text{max}}}p\left(v|m\right)p\left(m\right)\;\;, \end{array}$$ (1)

where *p*(*m*) is the probability that a candidate spends an amount of money *m* in the campaign and *m*_*max*_ is the maximum amount of money that can be spent (see Fig 1).

Eq (1) is the basis of Superstatistics for non-equilibrium systems [26]. This theoretical framework was developed to describe the thermostatistics of an ensemble of particles where the temperature fluctuates in space and/or time. The Boltzmann-Gibbs statistics assumes that all intensive quantities are invariant and so, the weight of a configuration is always the same. By contrast, in Superstatistics, since different particles are at different effective temperatures, the weight of a configuration depends on the effective temperature. Thus, all probabilities depend on the temperature distribution. In an election, the probability that a candidate obtains a certain number of votes is a function of the amount of money *m* spent in the campaign, being *m* the analogue for elections of the temperature in a thermal system. In the limit where all candidates spend the same amount of money *m*, the Boltzmann-Gibbs statistics should be recovered.

To calculate *p*(*v*|*m*), let us consider a proportional election with *N*_*c*_ candidates and *N*_*v*_ total votes. Based on the principle of maximum entropy [27], *p*(*v*|*m*) should maximize the Shannon entropy,

$$\begin{array}{c} S=-\sum_{i=1}^{N_{c}}\sum_{v=v_{0}}^{\beta m_{i}}p\left(v|m_{i}\right)\text{ln}\left[p\left(v|m_{i}\right)\right]\;\;, \end{array}$$ (2)

where *v*_0_ and *βm*_*i*_ are the minimum and maximum number of votes that the candidate *i* can receive, and *β* is a constant. For simplicity, hereafter we assume that *v*_0_ is the same for all candidates. At this point, two constraints need to be imposed, as both the number of candidates *N*_*c*_ and total votes *N*_*v*_ are fixed (see Fig 1). In this way, the first constraint is then,

$$\begin{array}{c} \sum_{i=0}^{N_{c}}\sum_{v=v_{0}}^{\beta m_{i}}p\left(v|m_{i}\right)=N_{c}\;\;, \end{array}$$ (3)

which ensures the normalization of *p*(*v*|*m*), while the second one is,

$$\begin{array}{c} \sum_{i=0}^{N_{c}}\sum_{v=v_{0}}^{\beta m_{i}}vp\left(v|m_{i}\right)=N_{v}\;\;. \end{array}$$ (4)

By maximizing *S* subjected to Eqs (3) and (4), we obtain

$$\begin{array}{c} p\left(v|m\right)=\frac{1}{Z(m)}e^{-\mu v}\;\;, \end{array}$$ (5)

where *Z*(*m*) is a normalization factor that depends on *m* and it is the analogue of the partition function in a thermal system, given by,

$$\begin{array}{c} Z\left(m\right)=\frac{e^{\mu (1-v_{0})}-e^{-\beta \mu m}}{e^{\mu }-1}\;\;, \end{array}$$ (6)

where *μ* is the Lagrange multiplier related to the second constraint (Eq (4)). Since the number of votes is limited, *p*(*v*|*m*) decays exponentially for *v* ∈ [*v*_0_, *βm*] and it is zero otherwise.

**Fig 2:**
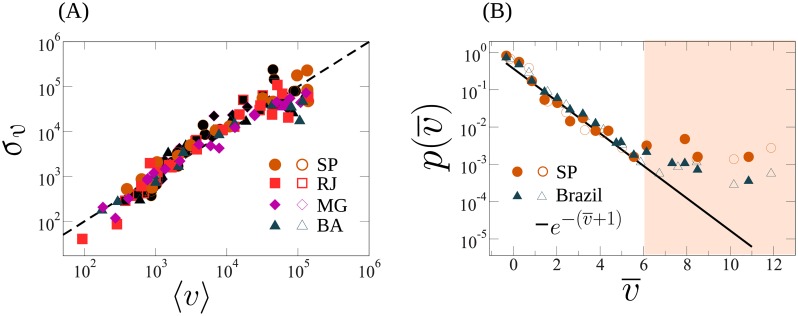
Empirical data for the 2014 and 2018 elections for federal deputies in Brazil, which counted more than 6000 candidates, roughly 140 million voters, and more than 280 million dollars of total investment in each campaign. (A) The standard deviation as a function of the average number of votes per candidate at the state level, for the top four populated Brazilian states, namely, São Paulo (SP, circles), Rio de Janeiro (RJ, squares), Minas Gerais (MG, diamonds), and Bahia (BA, triangles), empty symbols are for 2018, and filled symbols are for 2014. For each state, candidates were grouped by the amount of money that they officially declared to have spent in their campaigns. The dashed line corresponds to a linear behavior. (B) Distribution of rescaled number of votes $\bar{v}=\left(v-\langle v\rangle \right)/\sigma_{v}$ for São Paulo (orange circles) and the entire country of Brazil (blue triangles), where 〈*v*〉 and *σ*_*v*_ are the average and standard deviation of the number of votes received by the candidates in the same interval (logarithmic binning) of money spent. The black line corresponds to $p\left(v\right)=\exp\left(-\bar{v}-1\right)$, as predicted by Eq (5), if we assume *Z*(*m*) = 1/*μ*. Following the prediction given by Eq (5) from our theoretical approach, the distribution of votes for more than 99% of the candidates follows an exponential distribution. However, it is remarkable that the number of candidates with votes that deviate more than 6*σ*_*v*_ (highlighted region) from the average is higher than expected, suggesting the existence of outliers.

In order to verify if the distribution predicted by Eq (5) is compatible with real data, we consider the 2014 and 2018 elections for federal deputies in Brazil, using the dataset available in Ref. [28, 29]. Each state has its own ballot, with different candidates and voters. Countrywide, these elections had more than 6000 candidates each, roughly 140 million voters, and with over US $300 million investment in campaign. We first analyze the results for the top four populated Brazilian states, namely, São Paulo, Rio de Janeiro, Minas Gerais, and Bahia. These states have each more than 10 million voters and between 501 (Bahia) and 1686 (São Paulo) candidates for the 2018 election. For each state, we grouped the candidates by the amount of money that they reported to have spent in their campaigns. Fig 2A shows the standard deviation *σ*_*v*_ of the number of votes received by a candidate as a function of average number of votes 〈*v*〉 for each group. For most data point, the results are consistent with a linear behavior (dashed line) as expected for an exponential distribution, where the average and standard deviation are always equal. To verify the functional dependence of the distribution, in Fig 2B shows the distribution of votes, rescaled as $\bar{v}=\left(v-\langle v\rangle \right)/\sigma_{v}$, where 〈*v*〉 and *σ*_*v*_ is the average and standard deviation of the number of votes per candidate in the same interval (logarithmic binning) of money spent. The distribution clearly follows the predicted exponential behavior of Eq (5) for more than 99% of the candidates. However, for $\bar{v}>6$ the distribution deviates from the predicted one (highlighted region in Fig 2B). For 2014 there are eight candidates in this region in the entire country, all them running in São Paulo. This is remarkable, as the theory predicts only one in São Paulo. For 2018, there are eleven candidates for the entire country (six in São Paulo), although we would only expect seven. This observation raises doubts about these outliers and it could therefore call for a detailed analysis and validation of their reported data about the campaign founding.

From the partition function (6), the average number of votes received by a candidate that spent *m* money in the campaign is,

$$\begin{array}{c} \left\langle v\right\rangle =v_{0}+\frac{1}{e^{\mu }-1}+\frac{1-v_{0}+\beta m}{1-e^{\mu (1-v_{0}+\beta m)}}\;\;. \end{array}$$ (7)

**Fig 3:**
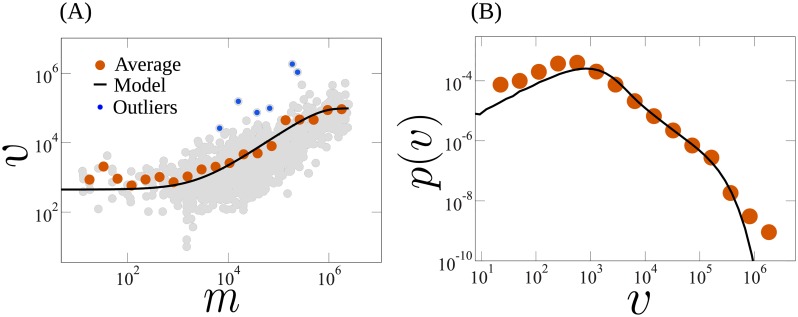
Results for the 2018 election for federal deputies in the state of São Paulo, Brazil, with 1686 candidates, more than 33 million voters, and about 29 million dollars of total investment in the campaigns. (A) Number of votes as a function of the money spent in the campaign for all candidates (gray circles) and the average value within each bin (orange circles). The (black-)solid line is obtained using Eq (7) with *β* = 0.609 (in units of inverse of money) estimated using the 2014 election (see Fig 4). The blue circles are outliers, which we defined as the candidates with a number of votes that deviate more than 6*σ*_*v*_ from the average. (B) Distribution of the number of votes per candidate. The (orange) circles were obtained from the data and the solid line was calculated from the distribution of money spent in the campaign. Precisely, the solid line is obtained by randomly assigning a number of votes *v* for each candidate from the distribution given by Eq (5), where *m* is the amount of money officially declared to have been spent in the campaign. The obtained curve is remarkably consistent with the empirical data over more than three orders of magnitude.

**Fig 4:**
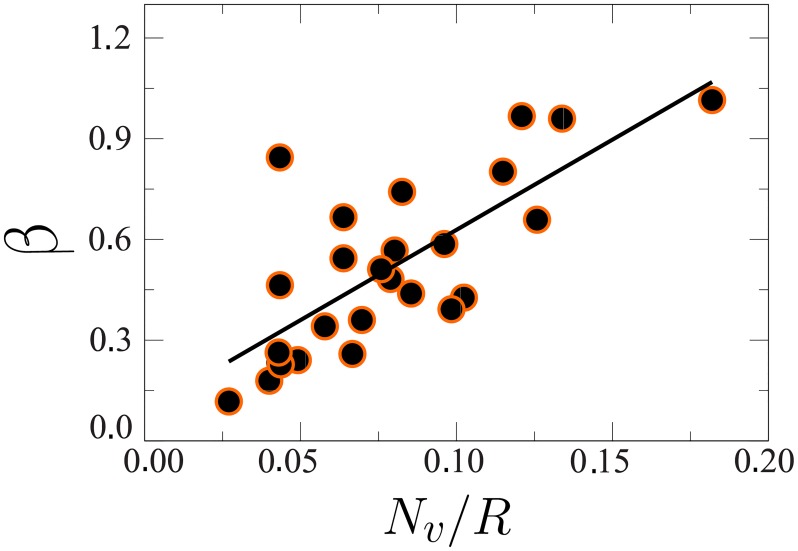
Parameter *β* as a function of the ratio of total number of votes *N*_*v*_ and money spent *R*. Results are obtained by fitting the data for the 2014 election for federal deputies in all states in Brazil with Eq (7). The solid line is a linear fit to the data obtained with the least-squares method.

The value of *μ* is obtained by imposing the second constraint (Eq (4)) and considering *β* as a free parameter. Fig 3A shows the number of votes per candidate against the money spent in the 2018 São Paulo campaign (gray circles) and the average value for candidates in the same money group (orange circles), where the circles in blue correspond to the outliers. To fit the data with Eq (7), one has one fitting parameter *β*. As shown in the Fig 4, *β* correlates strongly with the total money spent per voter in campaigns, so one can estimate *β* from the latter. As a proof of concept, we estimate the value of *β* for the 2018 election from the data for 2014, see Materials and Methods. For that, we assume a linear relation between *β* and the inverse of the total money spent per voter, parameterized using that data for 2014 (see Fig 4). The solid line in Fig 3A is the number of votes as a function of the money spent for the state of São Paulo in 2018 obtained with the estimated value of *β*. We observe an excellent agreement with the empirical data, that extends over four orders of magnitude. The deviation for candidates with very scarce resources can be explained as follows. For simplicity, we have considered that the minimum number of votes *v*_0_ is the same for all candidates, obtained by assuming that *v*_0_ equals the average number of votes for candidates who spent less than 1200 dollars [3]. In general, however, every candidate has a different *v*_0_, depending on several factors such as, his/her party, visibility, and social status.

From the predicted relation between *β* and the money spent per voter, we can also forecast the distribution of votes in 2018 using only the reported amount of money spent in this election, as shown in Fig 3B. More precisely, this is performed by assigning randomly a number of votes to each candidate from a distribution given by Eq (5), with *m* equal to the amount of money spent in the campaign, as declared by the candidate. The solid line in Fig 3B is the predicted outcome, which is in excellent agreement with the empirical data.

## Discussion

We have shown, using the principle of maximum entropy, that the distribution of votes received by a candidate should follow an exponential distribution parameterized by the amount of money that was spent in her/his campaign. This prediction is consistent with real data from a very large proportional election, with more than 6000 candidates. Furthermore, as the money spent in a campaign is heterogeneously distributed among candidates, we developed a framework based on superstatistics to establish the relation between the distribution of money spent and of votes. Within this framework, it was possible to predict the outcome of a ballot from the distribution of money spent, and identify potential cases of misconduct either in the report of fundraising and spending or on vote counting.

For several proportional elections, the distribution of votes per candidate is fat tailed [30], what has motivated an enthusiastic discussion about the underlying mechanism [10]. The fat tailed characteristic of the distribution of votes was first interpred as the result of a multiplicative process [8]. A different model was proposed based on world–of–mouth spreading for the case of proportional elections with open lists [19]. However, the empirical analysis performed in Ref. [30] showed that, although some countries yield similar distributions, the final shape of the distribution depends strongly on the specific election rules. Our theoretical approach shows, for an election, if all candidates spent the same amount of money in their campaigns, the expected distribution of votes would actually be exponential. So, the fat-tailed distribution is a consequence of an heterogeneous distribution of resources. This is consistent with the reported power-law distribution of money spent by candidates in the same elections [3].

## Materials and methods

### Electoral data

The data for the elections for federal deputies in Brazil in 2014 and 2018 were collected from the website of the Brazilian Superior Electoral Court [28, 29]. For each year, we analyzed two large datasets: the financial report of each candidate and the electoral results. The first dataset contains detailed information about the expenditures of all candidates. For each one, we calculated the total amount of money spent in the campaign by adding all their expenditures. The second dataset consists of the number of votes in each candidate for every electoral zone. We coarse grained this information, by adding all votes in the same candidate. By combining these two datasets, we obtained for each of the 26 Brazilian states, the list of candidates, the total amount of money that they spent in the campaign, and the final number of votes that they obtained. This adds up to 6353 and 7950 candidates, 87 million and 90 million votes, and 316 million and 335 million dollars spent in 2014 and 2018, respectively. The dataset is in the Supporting Information.

### An ensemble for elections

To determine *p*(*v*|*m*_*i*_) we maximize *W*, defined as,

$$W\;=\;-\;\sum_{i=1}^{N_{c}}\sum_{v=v_{0}}^{\beta m_{i}}p(v|m_{i})\;\text{ln}\left[p(v|m_{i})\right]\;-\;\lambda \;\left(\sum_{i=1}^{N_{c}}\sum_{v=v_{0}}^{\beta m_{i}}p(v|m_{i})-N_{c}\right)\;-\;\mu \left(\sum_{i=1}^{N_{c}}\sum_{v=v_{0}}^{\beta m_{i}}vp(v|m_{i})-N_{v}\right),$$ (8)

where the first term is the entropy, the second term is the constraint (3) with the Lagrange multiplier λ and the last term the constraint (4) with *μ* as the second Lagrange multiplier.

Imposing *dW*/*dp* = 0, we find that *p*(*v*|*m*_*i*_) = *e*^−1−*λ*−*μv*^ = *e*^−*μv*^/*Z*(*m*_*i*_). The expression for the partition function (6) is obtained by calculating $Z\left(m_{i}\right)=\sum_{v=v_{0}}^{\beta m_{i}}e^{-\mu v}$. From Eq (6), we obtain the average number of votes as

$$\begin{array}{c} \left\langle v\right\rangle =-\frac{\partial \text{ln}Z}{\partial \mu }=v_{0}+\frac{1}{e^{\mu }-1}+\frac{1-v_{0}+\beta m}{1-e^{\mu (1-v_{0}+\beta m)}}\;\;. \end{array}$$ (9)

In order to calculate the numerical value of 〈*v*〉 for each candidate, we first determine *μ*, by applying the constraint (4), where *μ* is the root of

$$\begin{array}{c} -\frac{N_{v}}{N_{c}}+v_{0}+\frac{1}{e^{\mu }-1}+\frac{1}{N_{c}}\sum_{i=0}^{N_{c}}\frac{1-v_{0}+\beta m_{i}}{1-e^{\mu (1-v_{0}+\beta m_{i})}}=0\;\;. \end{array}$$ (10)

This equation can not be solved analytically, therefore we used the SciPy implementation of the Brent’s method [31]. For 2014 election, we used the dataset of money expenditures during the campaign and the free parameter *β* was chosen as the value that minimizes the mean squared error between the votes expected value, Eq (7), and the real votes data.

To find the value of *μ* for 2018, we used the financial report of each candidate for that election. Since *β* correlates with *N*_*v*_/*R* (see Fig 4), we used the linear relation calculated to 2014 to estimate *β* for 2018.

### Data binning

To reduce the statistical noise in Figs 2 and 3, for each state, candidates were grouped by the amount of money that they officially spent in their campaigns. For that, we performed a logarithmic binning, limited by the minimum to the maximum amounts of money spent, always with 20 bins.

### Model for the distribution of votes

To forecast the distribution of votes in 2018 (Fig 3B), we considered the list of all candidates and the total amount of money spent in their campaign. For each candidate, we generated their number of votes at random, following the distribution derived in Eq (5), assuming *Z*(*m*) = 1/*μ*. In the limit *m* → ∞, we recover an exponential distribution *p*(*v*|*m*) = *μe*^−*μv*^.

The results in Fig 3B are averages over 10^4^ independent samples.

## Supporting information

S1 Table Data of 2014 election. The table is a comma separated file (CSV) with four columns: the state, the candidate number, the total money spent in Brazilian reais, and the total number of votes.
(CSV)

S2 Table Data of 2018 election. The table is a comma separated file (CSV) with four columns: the state, the candidate number, the total money spent in Brazilian reais, and the total number of votes.
(CSV)
